# Low Mortality of Orthopedic Trauma Patients With Asymptomatic COVID-19: A Level I Trauma Center Pandemic Experience

**DOI:** 10.31486/toj.21.0117

**Published:** 2022

**Authors:** Patrick A. Massey, Lincoln K. Andre, Steven M. Kautz, Chase Lobrano, R. Shane Barton, Kevin J. Perry, Brad J. Chauvin

**Affiliations:** Department of Orthopaedic Surgery, Ochsner Louisiana State University Health-Shreveport, Shreveport, LA

**Keywords:** *Coronavirus*, *orthopedics*, *pandemics*, *traumatology*

## Abstract

**Background:** Early (2020) reports on mortality in patients with coronavirus disease 2019 (COVID-19) who underwent orthopedic surgery ranged from 20.5% to 56%, but these studies included elderly patients with multiple comorbidities. The mortality rate for younger and asymptomatic COVID-19–positive patients undergoing orthopedic surgery after high-energy trauma is underreported. The purpose of this study was to compare the 30-day mortality of asymptomatic COVID-19–positive patients and COVID-19–negative patients surgically treated for orthopedic trauma at a Level I trauma center during the coronavirus pandemic. A secondary objective was to compare the patients’ postoperative hospital course and length of stay.

**Methods:** This study is a single-center retrospective review of all patients who underwent an orthopedic surgical procedure at a Level I trauma center during a 3-month period early in the COVID-19 pandemic. All patients received a preoperative nasopharyngeal swab to determine COVID-19 infection status. Preoperative demographic variables, perioperative and postoperative mortality within 30 days, length of stay, and intensive care unit days were compared between COVID-19–positive and COVID-19–negative patients.

**Results:** Of the 471 total patients, 13 were COVID-19–positive and 458 were COVID-19–negative prior to surgery. The average age of all patients was 40.5 ± 19.8 years. The mortality rate in the COVID-19–positive group was 0% vs 0.7% in the COVID-19–negative group, with no significant difference between groups (*P*=0.77). The COVID-19–positive group vs the COVID-19–negative group had no significant difference in hospital length of stay (7.4 days vs 4.4 days, respectively, *P*=0.12).

**Conclusion:** Asymptomatic COVID-19–positive orthopedic trauma patients treated with surgery at a Level I trauma center in a 3-month period during the COVID-19 pandemic had a 0% mortality rate, and we found no differences between COVID-19–positive and COVID-19–negative patients with respect to mortality and hospital length of stay.

## INTRODUCTION

The first case of coronavirus disease 2019 (COVID-19) in the state of Louisiana was reported on March 9, 2020.^1^ Louisiana was one of the first states to see an increase in coronavirus cases, resulting in fully occupied hospitals and scarce resources. During this time, regions of Louisiana had seropositive COVID-19 rates of 6.9%.^2^

Regional decisions were made to limit surgery selection based on hospital guidelines. Early data showed high mortality rates for COVID-19–positive patients undergoing surgery. One of the earliest reports from Wuhan, China, reported a mortality rate of 20.5% for elective orthopedic surgery.^3^ Subsequent research on COVID-19–positive patients treated for proximal femur and hip fractures showed mortality rates ranging from 21% to 56%.^4-6^ Many of the patients with hip fractures were elderly and had significant comorbidities. Further, while patients in their eighth decade can have a mortality rate of 22.8% with COVID-19 alone, patients in their fourth decade have a mortality rate <1%.^7^ The high risk of mortality for elderly patients with COVID-19 who sustain hip fractures has been established, but the mortality rate for younger high-energy trauma patients is underreported.

During the early stages of the pandemic, our hospital focused on emergent and urgent orthopedic surgeries, with rotations of teams on the trauma service to mitigate risk.^8,9^ Because our hospital is the only Level I trauma center within a 180-mile radius, our surrounding community hospitals absorbed the majority of elderly patients with hip fractures. Consequently, the majority of our COVID-19–positive patients were younger asymptomatic patients who were diagnosed on routine preoperative screening.

The purpose of our study was to compare the 30-day mortality of asymptomatic COVID-19–positive patients and COVID-19–negative patients surgically treated for orthopedic trauma at a Level I trauma center during a 3-month period early in the coronavirus pandemic. The secondary objective was to compare postoperative hospital course and length of stay between the 2 groups. Our hypothesis was that no difference in mortality would be seen between asymptomatic COVID-19–positive and COVID-19–negative orthopedic trauma patients treated with surgery at a Level I trauma center.

## METHODS

This retrospective study was approved by our institutional review board. Written informed consent was obtained from each patient before surgery per hospital policy. We conducted a medical record search of all trauma surgical procedures performed by our orthopedic surgery faculty from May 1, 2020, to July 31, 2020. These dates were chosen because prior to May 1, COVID-19 testing was inconsistent, but COVID-19 testing was consistent during the selected time frame. Also, because of limited resources and a surge in COVID-19 infections and hospitalizations, only emergency surgeries were allowed during this time period. These surgeries were nonelective procedures for patients presenting to our Level I trauma center. Patients were excluded if the procedure was a closed reduction of a fracture, although pediatric femur shaft hip spica casting was included because these patients undergo general anesthesia for this procedure at our institution.

During the study period, all surgical patients were required by hospital policy to have a COVID-19 test preoperatively. These tests were nasopharyngeal polymerase chain reaction (PCR) or rapid antigen detection COVID-19 test. COVID-19–positive patients were those who had a positive test within 5 days prior to surgery. Patients were determined to be asymptomatic COVID-19–positive if they had no fever, cough, or dyspnea upon admission with a positive COVID-19 test. COVID-19–negative patients were those who had a negative test prior to surgery or had a positive test with delay of surgery by more than 2 weeks, as these patients were considered negative by hospital protocol.

We reviewed patients’ medical records to obtain demographics such as age, race, sex, and body mass index (BMI); *International Classification of Disease*s*-10* diagnosis and Current Procedural Terminology codes for the surgical encounter; American Society of Anesthesiologists (ASA) physical status classification; hospital length of stay; days in the intensive care unit (ICU); and deaths within 30 days after surgery.

Numerical data such as ASA classification, ICU days, and age were compared between groups using a 2-sample *t* test. Categorical data such as race, sex, and type of surgery were compared using chi-square test.

## RESULTS

Initially, 634 surgical encounters were identified. Three patients who underwent closed reductions only were excluded, yielding 631 surgical encounters on 471 patients during the 3-month study period. Of the 471 patients, 13 were COVID-19–positive, and 458 were COVID-19–negative prior to surgery. During the study period, surgery was delayed for 2 patients because of a positive COVID-19 test. One patient had a chronic hip infection, and the other patient required a nerve repair; both surgeries were delayed by approximately 2 weeks, and these 2 patients are included in the COVID-19–negative group. Additionally, 2 patients who tested negative on admission tested positive for COVID-19 within 2 weeks after leaving the hospital. These 2 patients are also included in the COVID-19–negative group because they were negative at the time of surgery. These patients had no postoperative complications during the study period. All 13 COVID-19–positive patients had no fever or upper respiratory symptoms upon presentation and were labeled as asymptomatic COVID-19–positive.

Demographic data are summarized in Table 1. The average age of all patients was 40.5 ± 19.8 years and the mean ages of the COVID-19–negative and COVID-19–positive patients were similar (*P*=0.19). We found no difference in the proportion of males and females between groups (*P*=0.56), although the groups differed in racial distribution (*P*=0.02), with Black or African American patients comprising 84.6% of the COVID-19–positive group but only 41.0% of the COVID-19–negative group.

**Table 1.** Demographics and Comorbidities of Orthopedic Trauma Patients

Variable	All Patients, n=471	COVID-19–Negative Group, n=458	COVID-19–Positive Group, n=13	*P* Value
Age, years, mean ± SD (range)	40.5 ± 19.8 (2-91)	40.7 ± 19.9 (2-91)	33.4 ± 18.1 (2-61)	0.19
Sex				0.56
Male	306 (65.0)	296 (64.6)	10 (76.9)	
Female	165 (35.0)	162 (35.4)	3 (23.1)	
Race				0.02
White	256 (54.4)	255 (55.7)	1 (7.7)	
Black or African American	199 (42.3)	188 (41.0)	11 (84.6)	
Asian	0 (0)	0 (0)	0 (0)	
Other	13 (2.8)	12 (2.6)	1 (7.7)	
Unknown	3 (0.6)	3 (0.7)	0 (0)	
Body mass index, kg/m^2^, mean ± SD (range)	28.0 ± 7.9 (7.5-56.5)	28.1 ± 7.9 (7.5-56.5)	24.3 ± 6.4 (20.7-34.3)	0.10
ASA classification, mean ± SD (range)	2.2 ± 0.8 (1-5)	2.2 ± 0.8 (1-5)	2.4 ± 0.7 (1-3)	0.50
ASA classification				0.668
1	68 (14.4)	67 (14.6)	1 (7.7)	
2	242 (51.4)	236 (51.5)	6 (46.2)	
3	139 (29.5)	133 (29.0)	6 (46.2)	
4	21 (4.5)	21 (4.6)	0 (0)	
5	1 (0.2)	1 (0.2)	0 (0)	

Note: Data are presented as n (%) unless otherwise indicated.

ASA, American Society of Anesthesiologists.

For all patients, the average ASA classification was 2.2 ± 0.8, and the average BMI was 28 ± 7.9 kg/m^2^. The differences between groups for ASA classification and BMI were not significant (*P*=0.5 and *P*=0.1, respectively).

As shown in Table 2, no significant difference was noted in hospital length of stay, with a mean of 7.4 days for the COVID-19–positive group vs 4.4 days for the COVID-19–negative group (*P*=0.12). The average number of ICU days was higher for the COVID-19–positive group than the COVID-19–negative group (5.6 days vs 0.84 days, respectively, *P*<0.01). The mortality rate in the COVID-19–positive group was 0% vs 0.7% in the COVID-19–negative group, but the difference was not significant (*P*=0.77).

**Table 2.** Postoperative Course of Orthopedic Trauma Patients

Variable	COVID-19–Negative Group, n=458	COVID-19–Positive Group, n=13	*P* Value
Hospital length of stay, days, mean ± SD (range)	4.4 ± 6.7 (0-58)	7.4 ± 10.9 (0-36)	0.12
Intensive care unit admission			0.77
Yes	58 (12.7)	2 (15.4)	
No	400 (87.3)	11 (84.6)	
Intensive care unit stay, days, mean ± SD (range)	0.84 ± 3.1 (0-21)	5.6 ± 15.2 (0-18)	<0.01
Death			0.77
Yes	3 (0.7)	0 (0)	
No	455 (99.3)	13 (100)	

Note: Data are presented as n (%) unless otherwise indicated.

As shown in Table 3, the 471 patients had a total of 631 surgical encounters, with no difference in the types of primary surgeries performed in the COVID-19–positive vs the COVID-19–negative groups (*P*=0.271).

**Table 3.** Type of Orthopedic Surgery Performed and Side of Body

Procedure/Side of Body	COVID-19–Negative Group, n=458	COVID-19–Positive Group, n=13	*P* Value	CPT Code(s)
Amputation				
Above knee	1 (0.2)	0		27590
Finger	3 (0.7)	0		26951, 26952, 26910
Lower extremity fracture				
Acetabulum ORIF	14 (3.1)	0		27226, 27227, 27228, 27254
Pelvis ORIF	15 (3.3)	1 (7.7)		27215, 27216, 27218, 27217
Femur neck/proximal femur ORIF	5 (1.1)	0		27236, 27235, 27244, 27245
Pediatric femur hip spica	4 (0.9)	2 (15.4)		27502
Femur shaft/subtrochanteric ORIF	55 (12.0)	2 (15.4)		27506, 27507
Distal femur ORIF	2 (0.4)	1 (7.7)		27514, 27509, 27511, 27513, 27519
Patella ORIF	2 (0.4)	0		27524
Tibia plateau ORIF	19 (4.1)	1 (7.7)		27535, 27536
Tibia shaft ORIF	16 (3.5)	1 (7.7)		27756, 27758, 27759
Ankle ORIF	42 (9.2)	1 (7.7)		27766, 27769, 27792, 27814, 27822, 27823, 27829
Pilon ORIF	9 (2.0)	0		27827, 27828
Foot/calcaneus ORIF	9 (2.0)	0		28406, 28415, 28420, 28445, 28465, 28476, 28485, 28496, 28585, 28615
Removal of hardware, lower extremity	12 (2.6)	1 (7.7)		20670, 20680
Upper extremity fracture				
Clavicle ORIF	5 (1.1)	0		23515
Humerus shaft ORIF	23 (5.0)	0		24515, 27516
Distal humerus/elbow ORIF	12 (2.6)	0		24538, 24545, 24546, 24566, 24575, 24579, 24582, 24586, 24587, 24635, 24665, 24666, 24685
Elbow ligament repair/reconstruction	2 (0.4)	0		24343, 24344, 24345, 24346
Pediatric subcutaneous humerus pinning	4 (0.9)	0		24538
Forearm shaft ORIF	9 (2.0)	1 (7.7)		25515, 25525, 25545, 25574, 25575
Distal radius ORIF	26 (5.7)	0		25606, 25607, 25608, 25609
Hand/fingers fixation	18 (3.9)	0		26608, 26615, 26746, 26727, 26735
Removal of hardware, upper extremity	10 (2.2)	0		20760, 20680
Wound/infection				
Open fracture/wound upper extremity washout	58 (12.7)	1 (7.7)		20103, 11010, 11011, 11012
Open fracture/wound lower extremity washout	66 (14.4)	0		20103, 11010, 11011, 11012
Infection lavage and debridement upper extremity	25 (5.5)	0		11042, 11043, 11044
Infection lavage and debridement lower extremity	37 (8.1)	1 (7.7)		11042, 11043, 11044
Hip arthrocentesis (infection)	6 (1.3)	0		20610
Septic arthritis washout	4 (0.9)	0		23040, 23107, 24101, 24102, 26990, 26991, 27310, 29871
Arthroplasty				
Explant/hip antibiotic spacer	5 (1.1)	0		27091
Hip arthroplasty for femoral neck fracture	6 (1.3)	0		27236
Total hip replacement revision for infection or dislocation	10 (2.2)	0		27134, 27137, 27138
Total knee replacement revision for infection	5 (1.1)	0		27486, 27487, 27488
Damage control orthopedics				
External fixator removal	2 (0.4)	0		20694
External fixator upper extremity	1 (0.2)	0		20690, 20692
External fixator lower extremity	21 (4.6)	0		20690, 20692
Soft tissue				
Tendon repair upper extremity	29 (6.3)	0		24341, 25260, 25270, 26350, 26410, 26418
Tendon repair lower extremity	3 (0.7)	0		27385, 27650, 28200
Nerve repair upper extremity	5 (1.1)	1 (7.7)		64857
Fasciotomy	3 (0.7)	0		25020, 25023, 25024, 27602, 27894
Multiligament reconstruction for knee dislocation	3 (0.7)	0		27428, 27556, 27557, 27558, 29888, 29889, 27427, 27428, 27429
Tumor excision	4 (0.9)	0		21930, 26111, 27337,27365, 27075
Other	7 (1.5)	0		
Total surgical procedures	617	14	0.271	
Side of body			0.473	
Left	313	6		
Right	283	7		
Central	11	1		
Bilateral	10	0		

Notes: This table represents the number of primary surgical encounters. Some patients had return visits to the operating room with multiple surgical encounters. Data are presented as n (%).

CPT, Current Procedural Terminology; ORIF, open reduction internal fixation.

## DISCUSSION

Early research from 2020 in orthopedic patients indicated that COVID-19–positive patients had alarming mortality and morbidity rates in the perioperative and postoperative phases of care, including increased pulmonary complications, thromboembolic events, sepsis, cardiac events, and death.^4,6,10^ Although many of these studies focused on elderly patients or patients with low-energy hip fractures, these increased mortality and morbidity rates for COVID-19–positive patients in the perioperative period raised concerns and prompted calls for postponement or cancellation of elective cases and greater utilization of nonoperative fracture care.^11^

At our institution, while elective cases were postponed in accordance with state mandates, the orthopedic trauma service continued to operate on a variety of urgent and emergent injuries per a previously published protocol.^8^

Our study supports our hypothesis that no difference in mortality would be found between asymptomatic COVID-19–positive and COVID-19–negative orthopedic trauma patients treated with surgery at a Level I trauma center. We also found no significant difference in hospital length of stay, but we did see an increase in ICU days in the COVID-19–positive patients vs the COVID-19–negative patients. This finding is similar to the results reported by Lei et al who described a 44% postoperative ICU admission rate in a COVID-19–positive cohort of 34 patients.^3^ However, the absence of increased mortality in our COVID-19–positive patients is in contrast to some previous reports. Egol et al reported a mortality rate of 35.3% among 17 patients (mean patient age of 82.9 years) who underwent operative treatment of femoral neck fractures.^10^ LeBrun et al reported a 56% mortality rate in their cohort of 9 patients (mean patient age of 85 years, range of 65 to 100 years) who underwent surgery for hip fractures.^6^ Kayani et al reported a 30.5% mortality rate among 82 patients (mean patient age of 71.9 years) with hip fractures.^12^ The multicenter COVIDSurg Collaborative study reported a 28.8% 30-day mortality rate in 299 orthopedic operations (patient age ranged from <29 years to >70 years), although the type of surgery was not described.^13^ Several differences exist between our patient population and the populations reported in these studies. A key difference is that our patients had a mean age of 40.5 years, which is much younger than the patient ages reported in the studies summarized above.

Older age has been consistently identified as a risk factor for increased COVID-19 mortality in literature from around the globe.^14-16^ Additionally, the highest recorded mortality rates have been reported in the hip fracture population.^6,10^ Patients with hip fractures are generally older, have higher rates of medical comorbidities, and carry an increased risk of morbidity, as well as increased 30-day and 1-year mortality rates as a result of their injury.^17-20^ Hip fractures alone are associated with a high mortality rate, and elderly patients with COVID-19 have a high mortality rate, so elderly patients with a hip fracture and COVID-19 are likely to have a high mortality rate. This high mortality rate may not extend to younger orthopedic COVID-19–positive trauma patients, even those with severe injuries. Our Level I trauma center treated mostly high-energy trauma in a young patient population, which may explain the absence of mortalities in the COVID-19–positive group.

De et al conducted an observational study of 214 general orthopedic trauma patients in the United Kingdom and showed a 13% mortality rate among the patients with hip fractures and a 0% mortality rate in the population excluding the patients with hip fractures.^21^ De et al also showed that all deaths occurred in patients ≥70 years.^21^ Our patients underwent a variety of operations for acute trauma, but we had few proximal femur fractures. Our 0% mortality rate in COVID-19–positive patients may be explained in part by younger age and in part by our lack of patients with hip fractures. However, the baseline health of our patients may have also played a role in lowering overall mortality. Our COVID-19–positive patients had a mean BMI of 24.3 kg/m^2^. BMIs of 25 kg/m^2^ to 35 kg/m^2^ or greater have been linked to increased mortality, ICU admission, ventilator use, and need for hospital admission, particularly in a young population.^22-24^ The relationship between ASA classification and COVID-19 mortality is not clear, but some evidence suggests that our COVID-19–positive patient population, with a mean ASA classification of 2.4, may not have been at increased risk. Karayiannis et al reported on 484 orthopedic trauma patients with a COVID-19–positive mortality rate of 14.8%.^25^ They observed no mortalities in patients with ASA classifications 1 or 2. They also demonstrated a significantly increased risk of mortality for COVID-19–positive patients with an ASA classification of ≥3.^25^ The demographics of our trauma patient population are fairly representative of our service area in the South Central United States. Similar to previous studies, our COVID-19–positive cohort consisted of more Black or African American patients than any other race.^2^

Our study has several important limitations. First, our retrospective review using electronic medical records may not have captured all patients eligible for inclusion and therefore may have missed some COVID-19–positive patients. The most likely cause for this omission would be trauma patients transferred to our facility who presented with COVID-19 test results from an outside hospital that were not entered into our electronic medical record prior to surgery. Second, most of the patients identified as COVID-19–positive in our study were asymptomatic before surgery and during their hospital stay. Standardized protocols for COVID-19 testing were put in place in the early weeks of the peak spread to identify COVID-19–positive patients before proceeding to the operating room. Because all patients with and without symptoms were tested within the 5 days prior to surgery, our testing may have identified patients with a remote COVID-19 infection who had already recovered from the active stage of the disease. These patients would have been included in the COVID-19–positive cohort for our study but may not have carried the same risk as patients with active infection. Third, the single swab nasopharyngeal PCR testing and rapid antigen detection test protocol used in our study has some inherent weakness, with a sensitivity of approximately 86% and 70%, respectively.^26,27^

Also, we did not report how many patients at our institution received nonoperative management instead of surgery because of the COVID-19 pandemic. These decisions were made according to individual provider judgment of patient safety. The patients selected for nonoperative management possibly had higher rates of baseline medical comorbidities and were at higher risk for complications than the patients who underwent surgery. Not including these patients in our analysis may have led to an underestimation of the true risk of perioperative COVID-19 infection. However, we did not delay any surgeries that were urgent. Last, while our overall sample size was high, our number of COVID-19–positive patients receiving acute trauma procedures was low. Thus, our results are underpowered, with a high potential for type II error. However, our large overall sample of patients surgically treated during the COVID-19 pandemic provides useful data, and our sample size of COVID-19–positive patients is comparable to other published studies. Future meta-analyses should combine data from multiple centers to increase overall sample size.

Our study data indicate that younger COVID-19–positive orthopedic trauma patients in fair to good baseline health are at no increased risk of all-cause mortality in the perioperative and postoperative phases of care when undergoing surgical management of acute orthopedic trauma.

## CONCLUSION

The mortality rate among asymptomatic COVID-19–positive orthopedic trauma patients treated with surgery at a Level I trauma center during 3 months of the COVID-19 pandemic was 0%. We found no differences between COVID-19–positive and COVID-19–negative orthopedic trauma patients with respect to mortality and length of stay, but COVID-19–positive patients spent more days in the ICU. This study presents another piece of evidence for orthopedic surgeons who may be faced with weighing the risks and benefits of operating on a COVID-19–positive patient with acute orthopedic trauma.

## Notes

*This article meets the Accreditation Council for Graduate Medical Education and the American Board of Medical Specialties Maintenance of Certification competencies for Patient Care and Medical Knowledge.*
